# A Data-Driven Approach to Predict Fatigue in Exercise Based on Motion Data from Wearable Sensors or Force Plate

**DOI:** 10.3390/s21041499

**Published:** 2021-02-22

**Authors:** Yanran Jiang, Vincent Hernandez, Gentiane Venture, Dana Kulić, Bernard K. Chen

**Affiliations:** 1 Mechanical and Aerospace Department, Monash University, Melbourne, VIC 3800, Australia; Dana.Kulic@monash.edu (D.K.); Bernard.chen@monash.edu (B.K.C.); 2 Department of Mechanical Systems Engineering, Tokyo University of Agriculture and Technology, Tokyo 184-0012, Japan; vincent.hernandez1985@gmail.com (V.H.); venture@cc.tuat.ac.jp (G.V.)

**Keywords:** fatigue estimation, human motion data, deep learning, force plate, IMU, machine learning

## Abstract

Fatigue increases the risk of injury during sports training and rehabilitation. Early detection of fatigue during exercises would help adapt the training in order to prevent over-training and injury. This study lays the foundation for a data-driven model to automatically predict the onset of fatigue and quantify consequent fatigue changes using a force plate (FP) or inertial measurement units (IMUs). The force plate and body-worn IMUs were used to capture movements associated with exercises (squats, high knee jacks, and corkscrew toe-touch) to estimate participant-specific fatigue levels in a continuous fashion using random forest (RF) regression and convolutional neural network (CNN) based regression models. Analysis of unseen data showed high correlation (up to 89%, 93%, and 94% for the squat, jack, and corkscrew exercises, respectively) between the predicted fatigue levels and self-reported fatigue levels. Predictions using force plate data achieved similar performance as those with IMU data; the best results in both cases were achieved with a convolutional neural network. The displacement of the center of pressure (COP) was found to be correlated with fatigue compared to other commonly used features of the force plate. Bland–Altman analysis also confirmed that the predicted fatigue levels were close to the true values. These results contribute to the field of human motion recognition by proposing a deep neural network model that can detect fairly small changes of motion data in a continuous process and quantify the movement. Based on the successful findings with three different exercises, the general nature of the methodology is potentially applicable to a variety of other forms of exercises, thereby contributing to the future adaptation of exercise programs and prevention of over-training and injury as a result of excessive fatigue.

## 1. Introduction

Physical activity can improve health and well-being, reduce the risk of many diseases, and improve the quality of life [1]. However, a large number of people suffer injuries during exercise [2]. A major contributing factor of exercise injuries is fatigue [2]. Fatigue caused by repeated movement accumulates over time and may exceed the muscle tissues’ tolerance, contributing to musculoskeletal disorders (MSDs) [2,3,4]. Thus, monitoring and predicting fatigue are important to reduce the risk of injuries. In the context of sports training, fatigue estimation can be used by coaches and physical therapists to avoid high levels of fatigue, which may adversely impact training and hinder performance in competition. In the context of rehabilitation, many patients are instructed to perform rehabilitation exercises at home by themselves. Without the therapist’s instructions and feedback on their movements, there are greater risks of secondary injury. Therefore, actively monitoring the onset of fatigue could provide important feedback in sports training, competition, and rehabilitation [4].

Human activity recognition (HAR) is a broad research field that involves the identification of various human activities or gestures and more detailed knowledge about human activities (e.g., the quality of motions, emotions, and gender) based on sensor data [5,6,7]. Recently, many novel data processing and analysis methods have been applied to HAR due to the introduction of wearable, low cost, low power sensors and live streaming of data [5]. Meanwhile, advances in computer vision, machine learning, and artificial intelligence have enabled HAR to be widely used in athletic competition, healthcare, and elderly care applications [5,8,9,10]. Even though much research has been conducted on human movement analysis and action recognition [5,6,7], the study of automatic fatigue prediction or estimation is somewhat limited.

Typically, biomechanical variables are measured using motion capture for the kinematics of body segments, electromyography for muscle activity, and plantar pressure measurements for detecting stepping [11]. Common techniques for detecting fatigue are to measure muscle activity, e.g., surface electromyogram (sEMG) [12], and the kinematics of joint angles, e.g., optical motion capture [1]. However, sEMG has limitations, e.g., the sensors may lose contact over time, particularly during vigorous exercise, and can only measure the activity of the particular muscle to which the sensor is attached. For optical motion capture, limited capture area and occlusion are key issues because reflective markers can be hidden from the camera and additional performers will increase occlusion in team sports. Even though there are some technologies that use structural lighting [13] and multi-camera motion capture systems [14,15] that can exclude blind spots, they are difficult to apply to outdoor activities. The multi-camera system usually takes longer to set up due to the large amount of equipment and is less flexible. On the contrary, wearable IMUs are small, lightweight, and robust to occlusions and interference. They do not restrict body movements and allow a participant to perform various tasks in arbitrary environments. On the other hand, force plates (FPs) are easy to use and do not require any equipment on the body, therefore saving much time in the setup of experiments. They also help to record the motion abnormalities of lower body segments during specific activities [16]. Foot plantar sensors are also seldom discussed in the application of fatigue estimation. Therefore, force plates and IMUs are selected to measure exercise-induced fatigue, and their performances are compared.

A number of studies [17,18,19,20] have been conducted to detect binary fatigue (fatigue vs. non-fatigue) by using various invasive or noninvasive devices including IMUs and sEMG. However, these models can only represent a simplistic process of fatigue development, and an essential early intervention is impossible before the athlete is deemed to be fatigued. Limited research [1,20,21] has been conducted to monitor gradual and continuous changes in fatigue levels. The change from low levels of fatigue to high levels of fatigue is a continuous process, which may take place gradually, and the variations of human motion due to fatigue are fairly small during exercise, making it difficult to detect continuous changes in fatigue [17]. Moreover, the associated literature [17,18,19,20] only focuses on one task or exercise type (running only or jumping only) and lacks the ability to generalize the fatigue models to different exercises.

The onset of muscle fatigue is complex and may depend on personal fitness level, health conditions, types of exercise, and gender [8]. The observed changes in movement or muscle electrical activity may not be consistent among all individuals. In other words, the expression of fatigue is more likely to be person-dependent.

In this work, a data-driven approach is investigated to estimate the onset of fatigue using force plate or IMU measurements in three different exercises (squat, high knee jack, and corkscrew toe-touch). The proposed framework is the first of its kind to model the continuous increase in fatigue based on each single repetition (rep) of exercise and the first to be evaluated on multiple exercises, showing its potential generalizability. Unlike most previous studies, which focused on extreme fatigue and non-fatigue detection, here, the approach is to monitor the fatigue accumulated status with continuous feedback. In the field of human motion recognition, the deep learning based framework makes a great contribution to understanding the quality of exercise for rehabilitation in a continuous process, automatically, without specific domain knowledge.

## 2. Related Work

In this section, learning approaches for fatigue estimation are discussed.

Various research has been conducted to evaluate fatigue through data from different sensors and different methodologies, and the choice of sensors depends on the purpose or objectives of each study. Most studies (e.g., [12,22,23,24,25,26,27]) were conducted using surface electromyogram sensors (sEMG) to determine the physiological status of a muscle due to the activity. For example, Chattopadhyay et al. proposed an exhaustive set of features from the sEMG signals and analyzed the variability between subjects and between trials [25]. Dong et al. proposed a method to evaluate the overall fatigue of human body movement based on combined sEMG and accelerometer signals and introduced a “forgetting factor” and fatigue level fusion coefficient to combine different localized muscle fatigue estimates with the overall fatigue level [4]. However, sEMG may lose contact over time, particularly for dynamic exercise, and can only measure the particular muscle to which a sensor is attached. It cannot be used in the real world.

Inertial measurement units (IMUs) have also been used to analyze fatigue based on kinematic movement, especially in gait [18], running [19,20,28], and sprinting [29]. Those results indicated the capability of IMUs to provide reliable and accurate measurements of temporal parameters during exercise. The most relevant studies to our research are [19,20]. Buckley et al. predicted subject-dependent and subject-independent fatigue levels (non-fatigued and fatigue status) through data from a single IMU [19]. In the experiment, running four-hundred meters at a natural pace was considered as a non-fatigue status, and a subsequent beep test (also known as the “multi-stage fitness test”) was used to induce fatigue. The results showed that a single IMU on the right shank had better performance than on the lumbar spine when assessing subject-independent fatigue estimation, and the subject-dependent classifier had higher accuracy than the subject-independent classifier. Stohrmann et al. [20] monitored human fatigue by extracting kinematic parameters from wearable sensor data and investigated the kinematic changes evoked by fatigue during running. Twenty-one runners of different skill levels performed experiments on a treadmill and conventional outdoor track. Their findings showed that kinematic changes were related to fatigue for all runners, and fatigue was dependent on participants’ running technique [20]. To date, there are only a few studies focusing on continuous movement changes induced by fatigue, e.g., [1,20,21]. Ramos et al. presented a machine learning system to evaluate fatigue using electromyographic (EMG) and heart rate variability (HRV) measurements [21]. The approach showed a potential to implement a combination of a dimensionless (0–1) global fatigue descriptor to reflect the onset of fatigue.

Additionally, in the past research, a force plate was generally used to measure the postural stability performance [30,31], and some studies assessed counter-movement jump performance through the investigation of the ground reaction force-time profile [32,33,34]. The study [34] showed that fatigue due to the exercise of the calf-muscles of one leg could influence the body balance in the short term, and this can be measured by a force plate and an accelerator, which indicates the potential to distinguish human motion status through these two tools. In view of automatic recognition technology, different classifiers have been applied, such as the fuzzy logic (FL) classifier, the random forest classifier [19], the support vector machine (SVM) classifier [12,18], and the hidden Markov model (HMM) classifier [35]. However, these currently applied algorithms can only classify fatigue and non-fatigued status, and it it challenging to monitor a gradual and continuous changing of fatigue level. The methods also vary according to various exercises and muscle types. Different exercises may cause fatigue in different human body parts, and the measures of fatigue highly rely on the types of sensor and sensor positions. Thus, this paper aims to develop related algorithms to detect continuous fatigue changes and generalize fatigue models for various exercises.

## 3. Materials and Methods

**Figure 1:**
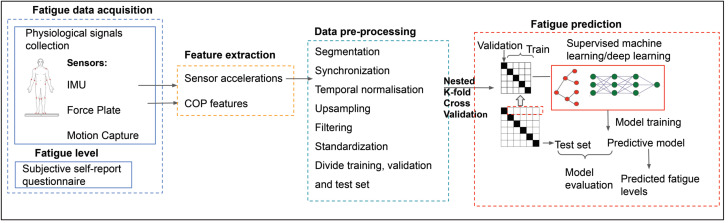
An overview of the proposed method. COP, center of pressure.

Subject-dependent recognition of fatigue for three different exercises, squat, high knee jack, and corkscrew toe-touch, is investigated. Figure 1 presents an overview of the four steps in the proposed methodology for predicting fatigue levels.

### 3.1. Data Collection

**Figure 2:**
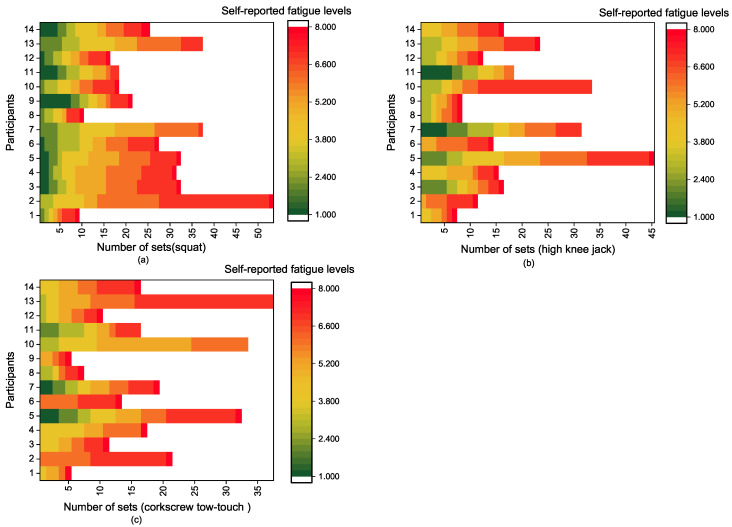
Distributions of the fatigue levels reported by participants in the experiment: (**a**) squat; (**b**) high knee jack; (**c**) corkscrew toe-touch.

**Figure 3:**
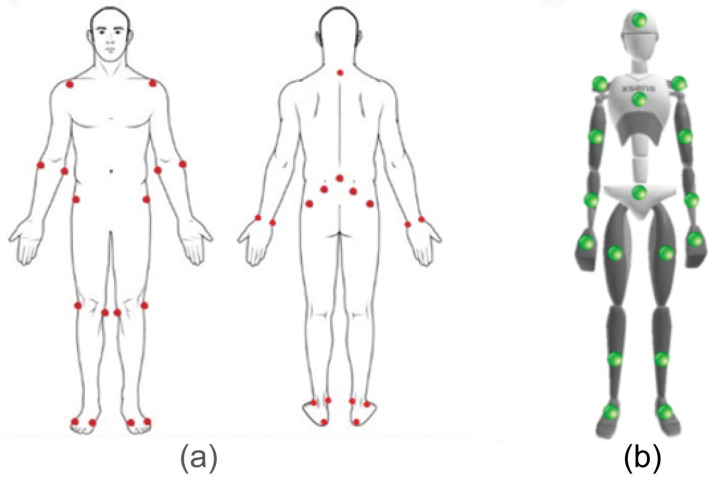
An illustration of the location of the markers. (**a**) Reflective markers for optical motion capture; (**b**) Xsens IMU sensors.

The fatigue dataset was collected using motion analysis motion capture (Motion Analysis, Santa Rosa, CA, USA) (sample rate: 100 Hz), Xsens IMUs (Xsens Technologies B.V., El Segundo, CA, USA) (sample rate: 240 Hz), and AMTI force plates (AMTI, Watertown, MA, USA) (sample rate: 100 Hz) simultaneously while performing full body exercises with one or two stationary feet. Figure 2 shows the distribution of the fatigue levels reported by participants in the experiment. A set of 32 reflective markers and 17 IMU sensors was attached on the human body (Figure 3). The dataset was collected at the University of Waterloo with ethics approval for the experiments. Fourteen healthy adults participated in the study (Table 1). The body weights were extracted from the force plate measurements. Before conducting the experiment, each participant was given a detailed explanation of the study and provided informed consent.

The participants were asked to perform three different exercises: squats, high-knee jacks, and corkscrew toe-touch, as described in Table 2. During each exercise, participants were asked to perform repeated sets of exercises to exhaustion. Each set consisted of 5 repetitions of an exercise. After each exercise set, participants were instructed to assess their fatigue level on a scale of 1 to 10 with “10” as “you are so tired you can’t stand up anymore”. The total number of sets of each exercise for each participant is summarized in Table 3, ranging from 3 to 52. This variation may depend on fitness level, age, gender, emotion, and pre-exercise fatigue level. Meanwhile, after each set, participants could take a short break to recover and then continue to the next exercise set.

### 3.2. Feature Extraction

The resultant accelerations and angular velocities were extracted from five IMU sensors on the lower body and selected as IMU features (sensors: right upper leg, right lower leg, left upper leg, left lower leg, and pelvis) for squat and high knee jack exercises. Since the corkscrew exercise also includes waist and hand motion, five sensors on the upper body were selected to measure their acceleration data (sensors: left upper arm, right upper arm, right forearm, left forearm, torso). In this study, the coordinates of the center of pressure (COP) were computed for a complete repetition according to Equations (1) and (2) and selected as input features instead of ground reaction force and moments (GRF&Ms). As the participant sways during exercise, the COP position varies over time and is one of the most commonly used features to quantify a person’s postural sway in both clinical and research contexts [36]. Comparing with the GRF&Ms, the COP features are more robust to inter-trial variability since they can remove the effect of the variability of different standing positions on the force plate. The complete feature set of the force plate used for training was the coordinates of COP and vertical ground reaction forces. Previous studies [36,37] have confirmed the reliability and informativeness of this choice of the feature set for postural stability.

(1) $$COP_{X}=\frac{-M_{Y}+\left(F_{X}\times d_{Z}\right)}{F_{Z}}$$

(2) $$COP_{Y}=\frac{-M_{X}+\left(F_{Y}\times d_{Z}\right)}{F_{Z}}$$

$F_{X}$, $F_{Y}$, and $F_{Z}$ respectively represent the components of the ground reaction force in which the *X* axis is in the mediolateral direction, the *Y* axis is in the anterior-posterior direction, and the *Z* axis in the superior-inferior direction. $M_{X}$ and $M_{Y}$ are components of the moments of force, and $d_{Z}$ is the depth of the force plate.

Other related COP features were also computed accordingly, including standard variance, variance, skew, kurtosis, area of the 95% ellipse, and first derivative and second derivative of COP positions. A explanation of the proposed features is provided in Table 4.

### 3.3. Data Preprocessing

Before using data in the conventional machine learning or deep learning model, it was necessary to preprocess the experimental data.

Segmentation: The continuous sequences of motion data obtained from the markers were divided manually into individual repetitions of squats by finding the segment points (i.e., the time step indicating the start and end of each repetition signal). Each repetition was labeled by the corresponding subjective fatigue level.

Synchronization: Since different sampling rates were used for the IMU data (240 Hz) and for the force data and marker data (100 Hz), a dynamic warping approach [38] was applied to synchronize the two different frequency time series data. This approach temporally aligns the two signals by identifying the alignment when the RMSE between two signals is minimized.

Temporal normalization: The lengths of the repetition segments were unevenly distributed due to the varying activity time of each participant. A fixed window size equal to 200 was considered, and each repetition was splined with a cubic spline interpolation.

Upsampling: Imbalanced datasets can skew the classifier towards the class that has the most samples [39]. Because only the beginning and end samples for each participant were in low and high fatigue while many samples were in between them, the highly unbalanced dataset was upsampled. The samples of each fatigue level were upsampled by adding additional samples to the minority class through random duplication, ensuring the final number of the minority class samples was equal to the number in the majority class.

Filtering: Butterworth low-pass filters with a cutoff frequency of 20 Hz were applied to all signals for noise reduction.

Standardization: The training, validation, and test data were standardized for each subject by computing the global mean μ and standard deviation σ from the training and validation sets. Then, each feature $x_{m,n}$ was standardized as follows:

(3) $$x_{m,n}=\frac{x_{m,n}-\mu_{norm}}{\sigma_{norm}}$$

Training, validation, and test sets: The dataset of each participant was separated into a training set, a validation set, and a test set, which composed approximately 70%, 15%, and 15% of the motion data, respectively [40]. Before splitting the training/validation/test sets, the data sorted by time order were randomly shuffled, ensuring that the training/validation/test sets were representative of the overall distribution of the data. Then, nested K-fold cross-validation with a user-dependent approach was performed on the dataset, which consisted of a rotating K = 5 folds in the inner loop and K = 6 folds in the outer loop. The inner loops were used to stop the training when the accuracy of the training set increased, but the accuracy of the validation set decreased in case of overfitting of the model on the training set [41]. Moreover, they were also used to tune the hyperparameters of the classifier (e.g., number of layers, cells unit size, learning rate, etc.). Five trained models were created after K = 5 fold cross-validation. In each outer loop, the held-back test set was fed to each model (K = 5), and the majority vote decision was applied to select the final predicted class. The final performance of the classifier was averaged over 6 outer loops.

### 3.4. Data Analysis

Two regression models were trained to predict fatigue: random forest (RF) regression and convolutional neural network (CNN) regression. Random forest is more robust to hyperparameters compared to other traditional machine learning algorithms and is also computationally efficient [42]. In this study, the random forest regression hyperparameters were the number of estimators and the depth.

Compared with the commonly used recurrent neural network (RNN) for time series data, the convolutional layers of the CNN can better capture local and temporal patterns because the main assumption of the CNN model is that the same local patterns are relevant everywhere [43]. CNNs also tend to be more computationally efficient because there are fewer sequential calculations. Both random forest and CNN models are trained based on repetition based predictions.

**Figure 4:**
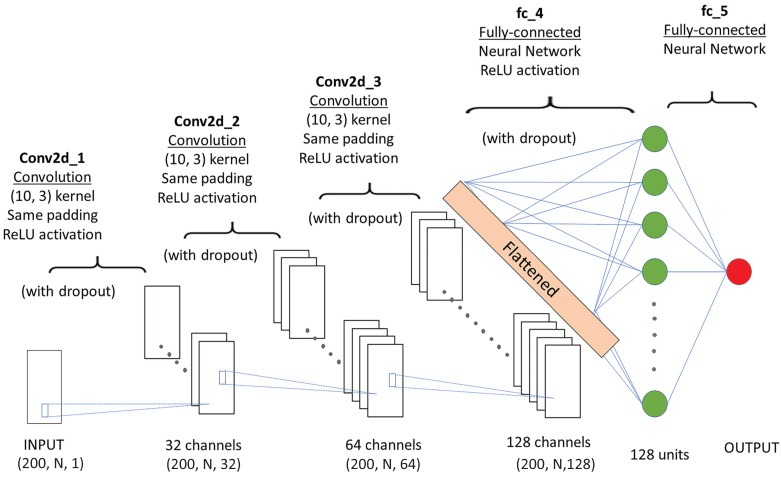
CNN structure (N represents the number of features. In this study, the value of N is 6 and 10 when using the force plate and IMU features as the input, respectively; 1, 32, 64, and 128 are the extracted channels at the corresponding convolution layer, and the stride size is [1, 1].).

The baseline CNN architecture has three 2D convolutional layers followed by a fully-connected layer (Figure 4). Rather than using hand-crafted features, the CNN directly learns features from the time series data without any prior knowledge of the features. Three convolutional layers were used to extract relevant features in the input signal. Each layer received the raw signals (or the one from the previous layer) and performed a convolution through a 2-dimensional kernel size of [10, 3] without pooling to extract relevant patterns. The filters for the three convolutional layers were 32, 64, and 128. The stride was [1, 1]. The rectified linear unit (ReLU) was employed as the activation function in the convolutional layers. To prevent overfitting, a dropout wrapper was added to each convolutional layer as a form of regularization to randomly select neurons (units) that were ignored at each epoch with a probability value of 0.8 [10]. Then, the output of the Conv layers was fed into a fully-connected layer composed of 128 units. Sigmoid was used as the activation function in the dense layer [10]. The Adam optimizer [44] was employed to minimize the cost function. The output layer had only one node. The CNN-based regression was trained with a batch size of 50 and a learning rate of η = 0.0001. The above hyperparameters were tuned by grid search. The model was trained by minimizing the loss function, where we used the root mean squared error (RMSE) between the predicted fatigue value and subjective fatigue level. The loss function (RMSE) is defined as:

(4) $$RMSE=\sqrt{\frac{\sum_{i=1}^{N}{\left(Predicted_{i}-Actual_{i}\right)}^{2}}{N}}$$

Pearson’s correlation coefficient ρ between the estimated level of fatigue and self-reported fatigue level was used as the evaluation criterion (as shown in Equation (5)). The coefficient ρ can be categorized as weak (ρ≤0.35), moderate (0.35<ρ≤0.67), strong (0.67<ρ≤0.9), or excellent (ρ>0.9) [45,46]. For each K-fold cross-validation, the training phase was stopped when the accuracy of the validation set started to decrease, and the corresponding CNN-based regression was saved.

(5) $$\rho =\frac{\sum_{i=1}^{n}\left(x_{i}-\bar{x}\right)\left(y_{i}-\bar{y}\right)}{\sqrt{\sum_{i=1}^{n}{\left(x_{i}-\bar{x}\right)}^{2}}\sqrt{\sum_{i=1}^{n}{\left(y_{i}-\bar{y}\right)}^{2}}}$$

where $x_{i}$ is the predicted fatigue level, $y_{i}$ is the actual fatigue level, and $\bar{x}$ and $\bar{y}$ are the related mean values.

## 4. Results

The experiments were performed with open-source deep learning libraries, TensorFlow-based Keras [47] and Scikit-learn [48]. The computer used for the experiments was equipped with 3.6 GHz i7-8700 processors and an NVIDIA GTX1060 GPU.

### 4.1. Comparison of Different COP Features

**Figure 5:**
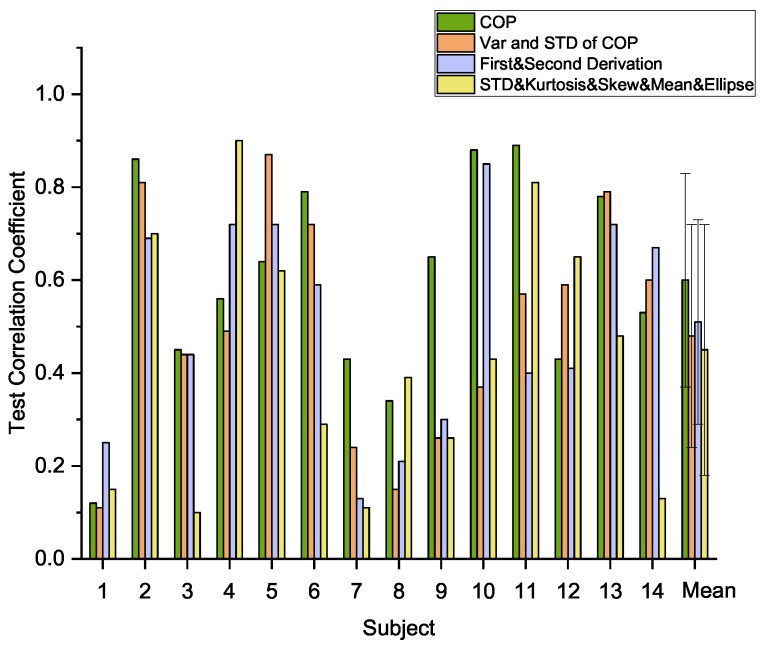
Correlation results with different COP features.

Since random forest is very sensitive to the selection of features, different feature groups were trained in the random forest regression model with squat motion data. The results are summarized in Figure 5. The average Pearson coefficients were determined for all subjects, and the first COP feature set (i.e., the coordinates of COP and vertical ground reaction force) was found to perform best compared with other COP features. A one-way analysis of variance with repeated measures was performed to determine if the first COP feature set and the other COP feature sets differed significantly in their performance for predicting fatigue levels for each participant. The statistics software SPSS for Windows, Version 25 (IBM Corp., Armonk, NY, USA), was used in the analysis. It showed a significant main effect (F(3,42)=7.928,p<0.05) when the significance level was set at α=0.05, which showed that the first COP feature set performed significantly better than other COP feature sets at this 0.05 level.

### 4.2. Performance of Fatigue Estimation with Only IMU Data vs. Only FP Data

**Figure 6:**
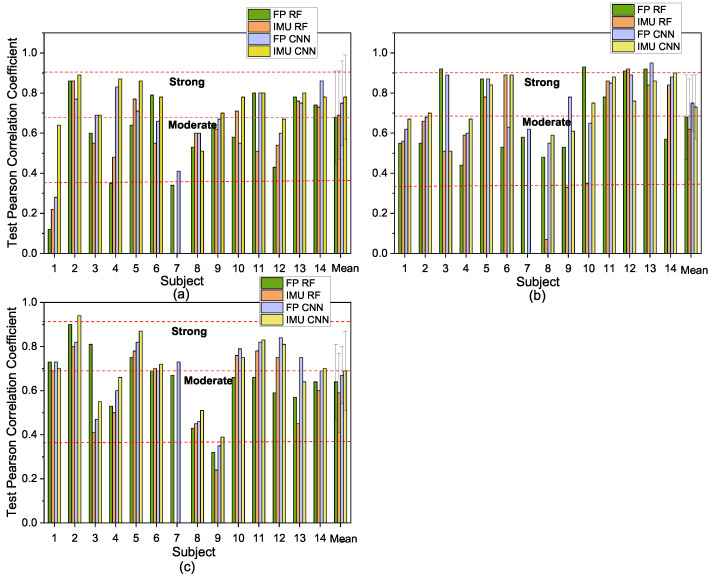
Results with IMU only and force plate (FP) only in the RF and CNN models: (**a**) squat; (**b**) high knee jack; (**c**) corkscrew toe-touch.

**Figure 7:**
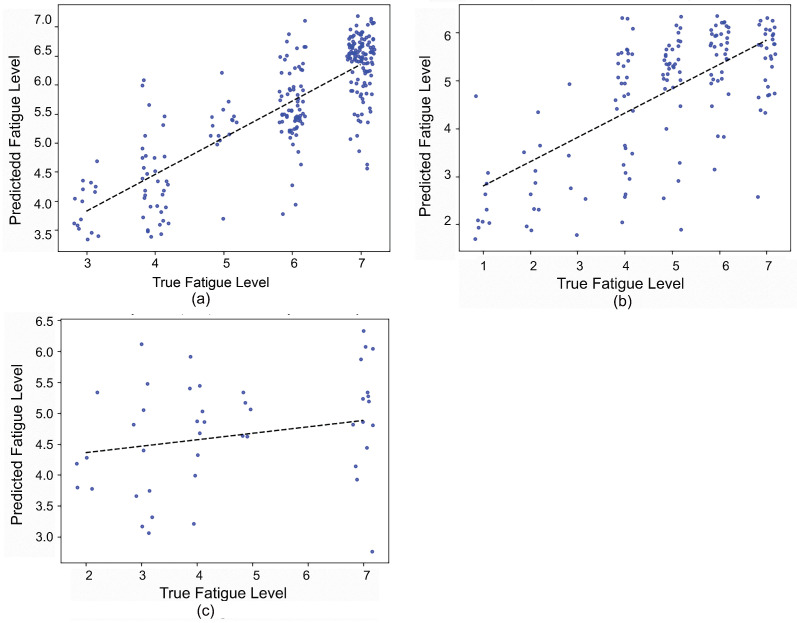
The predicted results of example subjects based on squat motion data from the FP representing the correlation between the predicted fatigue level and self-reported fatigue level (**a**) Subject 2 (strong correlation), (**b**) Subject 5 (moderate correlation), (**c**) Subject 8 (weak correlation).

The selected COP and IMU feature sets were applied to train the random forest and CNN models separately for three different exercises. Then, the selected and trained models after cross-validation were fed with the corresponding test set. In Figure 6, the test results of each participant with different models are compared for the squat, high knee jack, and corkscrew toe-touch exercises, respectively. Examples of the regression relationships between the predicted and true fatigue levels are shown in Figure 7 (Participants 2, 5, and 8 based on force plate data for the squat exercise).

Paired t-tests with different models showed that there was no significant difference between the performances of the FP and IMU for the test sets at the 0.05 level in each random forest model and CNN model using the motion data from the squat, high knee jack, or corkscrew toe-touch exercises. For the squat and high knee jack motion exercises, the CNN model performed significantly better than the RF model, with both FP data and IMU data, while for the corkscrew toe-touch exercise, the performance of both methods did not differ significantly.

**Figure 8:**
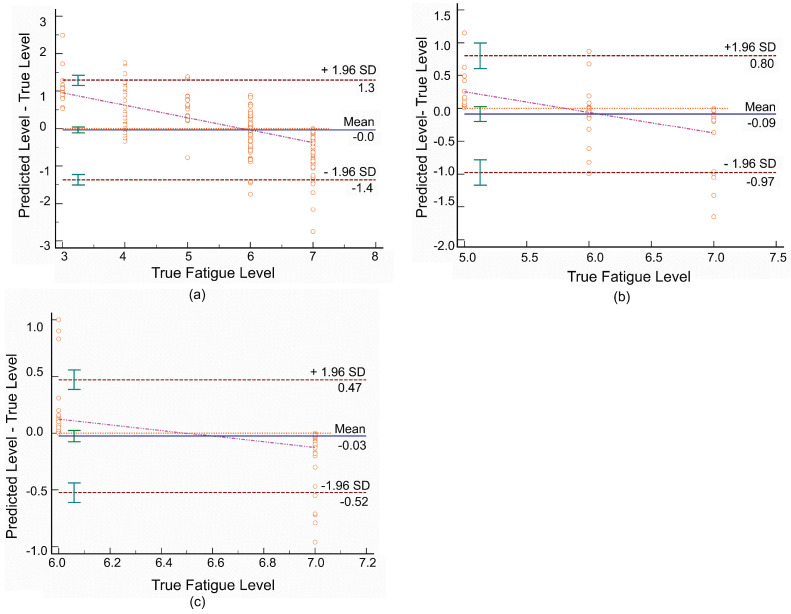
Example Bland–Altman (B&A) plot of a participant (subject = 5) with FP data for (**a**) squat, (**b**) high knee jack, and (**c**) corkscrew toe-touch. The blue line represents the mean differences. The red lines represent the 95% CI of the limits of agreement (the 1.96 SD). The pink line is the regression line of the differences. The orange dotted line is the line of equality (difference = 0).

A Bland–Altman (B&A) plot was generated [49] to describe the difference between true fatigue values and predicted fatigue values. It was found that the majority of test points were within the 1.96 standard deviation (SD) agreement, and the mean difference between predicted values and true values was close to zero. In other words, most of the predicted fatigue values were close to the true values for each participant. For participants who achieved a strong Pearson coefficient, the agreement limits in the B&A analysis ranged from approximately −0.5 to 0.5; whereas for participants with a moderate or a weak Pearson coefficient, the corresponding limits of agreements were from around −1 to 1 and −2 to 2, respectively. Figure 8 shows example B&A plots of a participant with FP data with a moderate Pearson coefficient in squat motion and a strong Pearson coefficient in high knee jack and corkscrew toe-touch motions.

## 5. Discussion

The aim of the present work was to evaluate the methodologies for monitoring continuous fatigue levels automatically during exercise. The performances of the random forest and CNN classifiers using force plate and IMU data were evaluated in this study. By comparing the correlation coefficient in the RF model, the displacements of COP were found to be most highly associated with fatigue during exercise than other commonly used features of COP including standard variance, variance, skew, kurtosis, area of ellipse, and the first derivative and second derivative of COP positions.

By applying the selected feature set of the force plate and IMU in a participant-specific prediction model for continuous fatigue, the results showed that motion data from the FP had similar performance for fatigue prediction as the IMU in terms of the average Pearson correlation coefficient. Up to an 89%, 93%, and 94% Pearson coefficient for the squat, jack, and corkscrew exercises, respectively, could be achieved for a subject-dependent regression model for continuous fatigue prediction with motion data from the force plate. The proposed model translated the participants’ perceived exertion into numerical scores ranging from zero to 10, contributing to quantifying the movement performance via supervised learning. In addition, for most participants, the CNN performed better than the RF regression model. As expected, since the CNN is capable of extracting features automatically and no specific domain knowledge is required, the results were more accurate than those of random forest. Participants 2, 5, and 13 were found to consistently achieve strong correlation between the estimated fatigue level and their self-reported fatigue level in the squat exercise. The same conclusion can be drawn for Participants 5, 11, 13, and 14 in the high knee jack exercise and for Participants 2, 5, 11, and 13 in the corkscrew exercise. Considering the data and demographics (Table 3), the prediction performance was highly related to the number of sets that participants performed. The larger the number of repeated exercises per subject, the higher the performance of the prediction was, while the participants who performed a few reps of exercises had weaker correlations in the predicted model. This can be explained as large training data are expected to improve the model performance of the prediction. A further analysis with the same amount data for each participant will be conducted to examine the predictions in future work. Furthermore, when the experiments last for a long time, participants may lose interest and feel bored rather than really feel tired. In order to complete the experiments quicker, they may exaggerate fatigue levels, leading to unreliable results and low accuracy [50]. Therefore, it may be necessary to consider this respondent fatigue during complex and long surveys or experiments.

Unlike most previous work, which has been limited to studies on fatigue during one type of exercise [1,17,18], the present study was conducted on three different exercises. The test Pearson correlation coefficients in the RF and CNN models with FP data or IMU data demonstrated a good generalization ability of the proposed methods for different exercises. Since fatigue takes place gradually in a continuous fashion, the present work addressed the limitations associated with assuming only a two-class fatigue level prediction [17,18,19,20,29], thereby capturing the buildup of fatigue during the progress of exercise, which is important for preventing over-training at the early stage of fatigue, contributing to reducing the risk of injuries. Our model not only can predict the onset of fatigue, but also provide insight into the gradual change of exercise performance due to fatigue.

The most related to our study is the study [1], which conducted experiments to quantify the person-dependent continuous increase of fatigue over time, based on a squat dataset recorded with optical motion capture. It predicted fatigue for sets of squats assuming the foregoing squats were performed with the same fatigue level as the last squat within a set, which fit well with the subjective fatigue ratings since the ratings were given after sets of squats. In contrast, here, predicted fatigue was for every single repetition of squats without a prior assumption. In terms of accuracy, the predicted fatigue level in our study was also close to the real level based on the B&A analysis. The deviation of the regression line of the difference was small, which shows that the model can achieve good prediction whatever the level. In addition, optical motion capture was used in [1], which would pose challenges in real-world applications, since the capture space is limited and it is difficult to apply to outdoor activities. The proposed approach requires manual segmentation of the motion repetitions. For online deployment, automated segmentation needs to be implemented [51,52]. Alternatively, the approach could be modified to consider fixed-size windows, so that segmentation is not required [53]. Based on the successful findings of this study, there is potential to implement a real-time application for monitoring continuous fatigue-induced changes of motion based on data from wearable sensors or wireless insoles. In the future, the authors will explore the influence of sensor positions on movement detection across multiple exercises and a participant-independent approach for fatigue estimation by collecting a large-scale dataset through simulation techniques.

## 6. Conclusions

- In this paper, a data-driven approach was presented for predicting fatigue based on data from IMUs or a force plate using random forest and the CNN.
- A high correlation (Pearson coefficient of up to 89%, 93%, and 94% for the squat, jack, and corkscrew exercises, respectively) can be achieved for a subject-dependent regression model for continuous fatigue prediction.
- The proposed model can detect fairly small variations of human motion due to fatigue, which also indicates the ability to monitor the gradual decline in the quality of the movement during exercise execution. The results contribute to the area of human motion recognition for more detailed knowledge of human motion.
- Fatigue prediction using force plate motion data had a similar performance as the data from IMU. The displacement of COP was found to be correlated to fatigue compared with other commonly used features of the force plate. Bland–Altman analysis also confirmed that the predicted fatigue levels were close to the true values.
- These results show that it is feasible to predict the onset of fatigue and quantify continuous fatigue levels from IMU data or force plate data through machine learning and deep learning.
- Based on the successful findings with three different exercises, the general nature of the methodology is potentially applicable to a variety of other forms of exercise and developing real-time biofeedback applications based on kinematic or kinetic data, thereby contributing to future adaptation of exercise programs and prevention of over-training and injury as a result of excessive fatigue.

## Figures and Tables

**Table 1:** Participants’ characteristics (SD: standard deviation).

Characteristics	Range				
	Number	%	Min	Max	Mean (SD)
Total Number	14				
Female	2				
Male	12				
Age (years)			20	35	27.4 (4.15)
Anthropometric					
Height (m)			1.62	1.87	1.76 (0.076)
Weight (kg)			57	99	74.13 (12.13)
Exercise hours per week (h)			0	12	4.53 (3.44)
0	2	13.3			
[1,5)	6	53.8			
[5,10)	3	26.7			
>10	2	13.3			

**Table 2:** Exercise descriptions.

Name	Initial Pose	Description
Squat	Standing	Bend knees to lower torso vertical position while keeping upper body upright and head forward
High knee jack	Standing	Start (arms above head; legs straight), ext1(right knee raise; arm comes down so hand claps under the leg), return to rest, ext2 (left knee raise; arm comes down so hand claps under leg)
Corkscrew toe-touch	Standing	Start (arm straight out to the side), ext1 (bend at waist, right hand touching left leg), return to rest, then ext2 (bend at waist, left hand touching right leg)

**Table 3:** Number of recorded exercise sets.

Participant	1	2	3	4	5	6	7	8	9	10	11	12	13	14
No. of sets of squat	9	52	31	30	31	26	37	9	20	17	20	15	37	24
No. of sets of high knee jack	6	10	15	14	44	13	31	7	7	32	21	11	22	15
No. of sets of corkscrew toe-touch	4	20	10	16	31	12	18	6	4	32	15	9	36	15

**Table 4:** Explanation of generated features.

Number	Name	Explanation
1	COP	The displacements of the center of pressure of two feet during each repetition
2	Variance of COP	Deviation of COP velocities in anteroposterior (AP) and mediolateral (ML) directions during each repetition
3	STD of COP	Standard deviation of COP velocities in AP and ML directions during each repetition
4	Mean velocity	Mean velocity, which represents the total distance traveled by COP for each repetition in AP and ML directions
5	First derivative of COP	The change rates of COP displacements (i.e., velocity) for each repetition in AP and ML directions
6	Second derivative of COP	The change rates of COP velocities (i.e., acceleration) for each repetition in AP and ML directions
7	Skew of COP	The value of skewness of COP displacements for each repetition in AP and ML directions
8	Kurtosis of COP	The value of kurtosis of COP displacements for each repetition in AP and ML directions
9	Ellipse of COP	The area of the ellipse covered by the trajectory of the COP for each repetition with a 95% confidence interval

## Data Availability

No new data were created or analyzed in this study. Data sharing is not applicable to this article.
